# Homocysteine Level and Risk of Hemorrhage in Brain Arteriovenous Malformations

**DOI:** 10.1155/2021/8862299

**Published:** 2021-03-30

**Authors:** Chaofan Zeng, Fa Lin, Peicong Ge, Dong Zhang, Shuo Wang, Jizong Zhao

**Affiliations:** ^1^Department of Neurosurgery, Beijing Tiantan Hospital, Capital Medical University, No. 119 South Fourth Ring Rd West, Fengtai District, Beijing 100070, China; ^2^China National Clinical Research Center for Neurological Diseases, Beijing 100070, China; ^3^Center of Stroke, Beijing Institute for Brain Disorders, Beijing 100070, China; ^4^Beijing Key Laboratory of Translational Medicine for Cerebrovascular Disease, Beijing 100070, China; ^5^Savaid Medical School, University of the Chinese Academy of Sciences, Beijing, China

## Abstract

**Objective:**

We aimed to investigate the risk factors associated with hemorrhage and clarify the relation of homocysteine (Hcy) with brain arteriovenous malformations (bAVMs).

**Method:**

We retrospectively reviewed bAVM patients from Beijing Tiantan Hospital between January 2019 and December 2019. Clinical and laboratory variables were analyzed in enrolled patients with bAVMs. Potential predictors associated with hemorrhage were evaluated by logistic regression analysis.

**Results:**

A total of 143 bAVM patients were identified in the study, including 69 unruptured and 74 ruptured cases. Patients with hemorrhage were less likely to have hyperhomocysteinemia (*P* = 0.023). Logistic regression analysis showed that increased maximum diameter of bAVM lesions (odds ratio (OR) 0.634, 95% confidence intervals (CI) 0.479-0.839; *P* = 0.001) and serum Hcy level (OR 0.956, 95% CI 0.920-0.993; *P* = 0.021) were associated with lower risk of hemorrhage in bAVMs.

**Conclusion:**

The present study provided evidence regarding the association between serum Hcy and hemorrhage in patients with bAVMs. Higher Hcy level was correlated with a lower risk of rupture. Detection of factors for subsequent hemorrhage is necessary to develop therapeutic strategies for bAVMs preferably.

## 1. Introduction

Brain arteriovenous malformations (bAVMs) are congenital collection of dilated arteries and draining veins without capillary beds, forming high-flow arterial blood shunts directly into the venous system [1, 2]. Intracranial hemorrhage manifests as the most common onset symptom with an annual rupture rate ranged from 2%-4%, which results in a high incidence of neurological morbidity and mortality [3, 4]. Although our understanding of bAVMs has progressed in recent years, causal relationships and the mechanisms by which bAVMs initiation, progression, and rupture remain poorly elusive.

Homocysteine (Hcy) is a nonessential amino acid from the methionine and folate cycle, a metabolic product critical for numerous biochemical processes. Clinically, normal serum level ranges from 5-10 *μ*mol/L, and incremental elevation above 15 *μ*mol/L is termed as hyperhomocysteinemia (HHcy) [5]. Previous studies and analyses suggested the atherogenic, thrombogenic, and procoagulant effects of Hcy [6, 7], which overlap the acknowledged pathogenic mechanism of bAVMs [8]. The major mechanisms were summarized as intracranial vascular injuries [9, 10], vascular wall remodeling [11, 12], hemodynamic stress [11], and diameter and hematoma expansion [13, 14]. Elevated serum Hcy is emerging as an independent risk factor for slow-flow occlusive cardiovascular diseases, neurodegenerative diseases, and ischemic stroke [6, 15]. However, after McEvoy et al. first presented the relationship between Hcy and hemorrhagic stroke [16], its role in hemorrhagic stroke subsequently reported remains controversial [17–19], let alone in bAVMs. Currently, most studies, including meta-analyses, failed to confirm this association [20–22].

To the best of our knowledge, no clinical study with relevant evidence that correlates Hcy with bAVMs has been performed so far. Thus, in the present study, we conducted this untargeted analysis to explore the risk factors associated with initial rupture and to clarify the relation of Hcy with bAVMs.

## 2. Materials and Methods

### 2.1. Study Design and Participants

We retrospectively reviewed patients diagnosed with bAVMs at the Department of Neurosurgery, Beijing Tiantan Hospital from January 2019 to December 2019. This study was approved by the Institutional Review Board of our institution. Informed consent was waived considering the retrospective design of the study.

**Figure 1:**
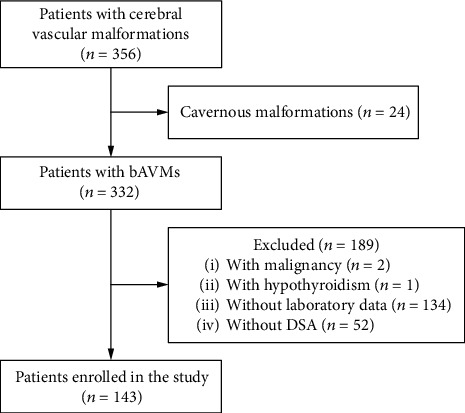
Flow diagram of the study participants. bAVMs: brain arteriovenous malformations; DSA: digital subtraction angiography.

Among 356 patients with cerebral vascular malformations admitted to our hospital between January 2019 and December 2019, 332 patients were diagnosed with bAVMs using magnetic resonance imaging (MRI) or digital subtraction angiography (DSA). Patients were excluded as follows: (1) with malignancies, chronic renal insufficiency, hematological diseases, or hypothyroidism; (2) with inadequate laboratory or DSA data. Finally, 143 patients were included in the study (Figure 1).

### 2.2. Data Collection

Demographic data, medical and operative history, clinical manifestations, bAVM characteristics, and laboratory results were obtained. Medical history including hypertension, diabetes mellitus, hyperlipidemia, cigarette smoking, and alcohol drinking were obtained. The history of prior treatment included endovascular embolization, radiosurgery, and microsurgery. Initial manifestations were summarized into three categories: hemorrhage, seizure, and neurological dysfunction. BAVM characteristics included size of lesion, eloquent and deep location, venous drainage, and associated aneurysms. The AVM volume was calculated by (a × b × c)/2 [23], and the Spetzler-Martin (SM) grading scale was evaluated to classify the bAVMs. Two experienced investigators (C.Z. and F.L.) independently reviewed the MRI and DSA images. Neurological status was assessed using the modified Rankin Scale (mRS) score at admission.

Heart rate and blood pressure were also recorded. Body mass index (BMI) was calculated as weight (kg)/[height (m)]^2^. Besides, fast venous blood samples were collected in the morning after admission for all patients. Levels of blood glucose, albumin (ALB), creatinine, uric acid (UA), total cholesterol (TC), triglyceride (TG), high-density lipoprotein cholesterol (HDL-C), low-density lipoprotein cholesterol (LDL-C), apolipoprotein A (ApoA), apolipoprotein B (ApoB), and homocysteine (Hcy) were measured using enzymatic methods. Serum Hcy ≥ 15.0 *μ*mol/L was considered as HHcy.

### 2.3. Statistical Analysis

All statistical analyses were performed using SPSS (version 26.0, IBM). The categorical variables were presented as frequencies, and continuous variables were described with mean (standard deviation, SD) or median (interquartile range, IQR). The chi-square test or Fisher exact test was conducted to compare categorical variables between groups. Continuous variables were compared using the two-tailed Student-*t* test or Mann–Whitney *U* test. The association between characteristics and Hcy quartiles was assessed using the Cochran-Armitage test for bivariate variables and Spearman's rank correlation test for continuous variables. Logistic regression analysis was performed to identify the risk factors for hemorrhage of bAVMs. Odds ratios (ORs) were calculated with 95% confidence intervals (CIs). Further exploration of Hcy associated with hemorrhage was conducted by adjusted models. *P* value < 0.05 indicated statistical significance.

## 3. Results

### 3.1. Clinical and Laboratory Characteristics of bAVM Patients

Baseline characteristics according to the hemorrhagic manifestation were shown in Table 1. A total of 143 patients with bAVMs were enrolled in the study. Compared with unruptured participants, patients with ruptured bAVMs tended to exhibit poor neurological status (mRS > 2) and less likely presented with seizure (*P* < 0.001 for both). Although the SM grade was found no difference between groups (*P* = 0.149), the average size of bAVMs was smaller in ruptured patients than in those without hemorrhage (*P* < 0.001). In addition, ruptured bAVMs showed lower levels of UA (*P* = 0.032). HHcy was found in 28 (40.6%) unruptured patients and 17 (23.0%) ruptured patients (*P* = 0.023).

### 3.2. Clinical Characteristics of bAVM Patients according to Hcy Quartiles

Analysis of the clinical characteristics according to Hcy quartiles was summarized in Table 2. A relationship was observed between Hcy level and male sex (*P* < 0.001). Serum Hcy level was also correlated with age (*P* = 0.005) and cigarette smoking (*P* = 0.007). Furthermore, Hcy was related to seizure manifestation and poor neurological status (*P* < 0.05 for all). Although the incidence of hemorrhage was no significant difference between Hcy quartiles (*P* = 0.093), a relatively lower risk of rupture occurred in the groups of higher Hcy concentration (Q1: 58.3%; Q2, 57.1%; Q3, 52.8%; Q4, 38.9%). The SM grade and other variables were similar across Hcy quartiles (*P* > 0.05 for all).

### 3.3. Analysis of Predictors for Hemorrhage of bAVMs

Predictors for the hemorrhagic presence of bAVMs were analyzed (Table 3). Univariate analysis showed that the maximum diameter of bAVM lesions and level of serum Hcy were associated with hemorrhage. After incorporating the covariables of age, male sex, smoking, prior embolization, deep location, eloquent location, deep venous drainage, and associated aneurysms into the multivariate analysis, maximum diameter (OR 0.634, 95% CI 0.479-0.839; *P* = 0.001) and serum Hcy level (OR 0.956, 95% CI 0.920-0.993; *P* = 0.021) were shown to be significantly related to hemorrhage.

**Figure 2:**
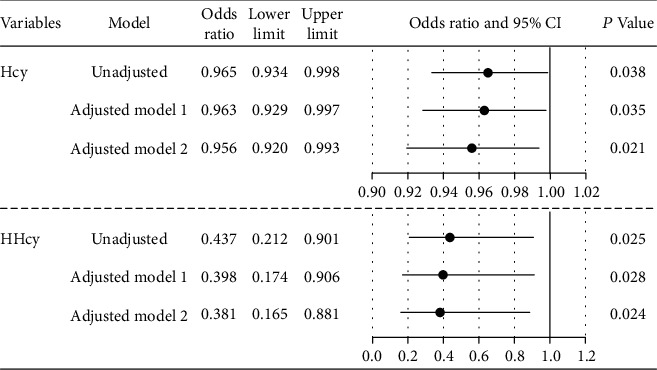
Odds ratio for hemorrhage in bAVMs, according to Hcy and HHcy. Model 1: adjusted for age, sex, cigarette smoking, eloquent location, deep venous drainage, maximum diameter of lesions, and Hcy. Model 2: additionally adjusted for prior treatment of embolization, deep location, and associated aneurysm. CI: confidence intervals; Hcy: homocysteine; HHcy: hyperhomocysteinemia.

**Figure 3:**
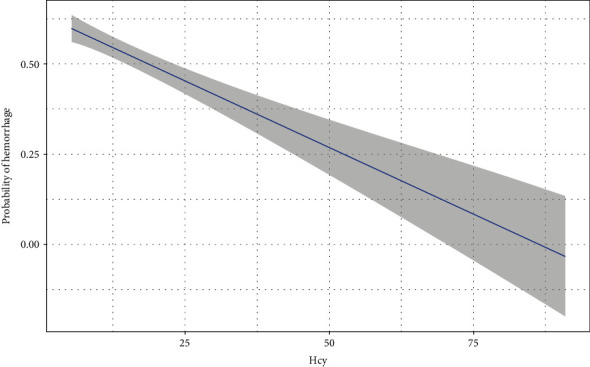
A linear relationship between Hcy level and probability of hemorrhage in the regression model. Hcy: homocysteine.

Prior to adjusting for potential covariables (Figure 2), HHcy was associated with the hemorrhagic presence (OR 0.437, 95% CI 0.212-0.901; *P* = 0.025). After adjusting for age, male sex, smoking, prior embolization, deep location, eloquent location, deep venous drainage, and associated aneurysms, HHcy was correlated with a lower risk of hemorrhage (OR 0.381, 95% CI 0.165-0.881; *P* = 0.024). The curve of regression model was fitted to reveal the linear relationship between Hcy level and probability of hemorrhage (Figure 3).

## 4. Discussion

In this retrospective study, we identified the potential risk factors for hemorrhage of bAVMs and investigated the association between serum Hcy level and bAVMs' rupture. HHcy was observed significantly less in patients with the hemorrhagic presentation. Furthermore, we found that increased size of bAVM lesions and higher levels of Hcy were correlated with a lower risk of hemorrhage.

Although HHcy was proved to be an independent risk factor for ischemic stroke [24], the relationship between intracranial hemorrhage and HHcy has not been extensively studied. A previous study involving 503 patients with ICH reported that the risk of hemorrhage in patients with HHcy was 1.94-fold compared to the controls [17]. A recent meta-analysis showed that Hcy levels in patients with ICH were significantly higher than in healthy participants [19]. Various diseases are characterized by intracranial hemorrhage, including hypertension, cerebral amyloid angiopathy, intracranial aneurysms, and bAVMs. Wang et al. reported that HHcy was an independent risk factor for the formation of intracranial aneurysms in the Chinese Han population [25]. HHcy may also be associated with the rupture of intracranial aneurysms in animal experiments [12]. According to the study by Kumar et al., higher serum Hcy level was observed in SAH patients as compared to healthy controls [26]. However, other studies demonstrated the contrary results. Dhandapani et al. revealed that the increased Hcy was significantly associated with favorable outcomes following SAH [27]. Moreover, a lower concentration of Hcy was verified as the predictor for hematoma expansion of ICH in cerebral small vessel diseases [28].

In our study, Hcy was proved to be associated with rupture in bAVM patients. Surprisingly, ruptured bAVMs had less proportion of HHcy, and the level of Hcy was an independent protective factor for hemorrhage. Hcy is an amino acid involved in the metabolism of methionine and folate cycle. The permeability of cerebrovascular endothelial cells is increased with MMP-9 (matrix metalloproteinase-9) activated by Hcy, accompanied with blood-brain barrier (BBB) destruction [29]. It has been found that Hcy may further activate MMP-9 through extracellular signal-regulated kinase (ERK) pathway and inhibition of *γ*-aminobutyric acid (GABA) receptor in endothelial cells [30]. MMP-9 may also disrupt the BBB by degrading type IV collagenase, laminin, and fibronectin in extracellular matrix and basement membrane; activating vascular endothelial growth factor (VEGF) and thrombin; inducing apoptosis, which exacerbates the occurrence of ICH [31]. Furthermore, increased levels of MMP-9 were observed in bAVMs. The abnormal expression of MMP-9 destabilized the vessels, which potentially leads to the rupture of bAVM lesions [32].

Alternatively, the results may be associated with hemodynamic abnormalities. Recent studies have documented the effects of Hcy on arterial hemodynamics [33–35]. Several lines of evidence suggested a converse relationship between Hcy and flow velocity for the coronary arteries [33, 35]. The elevated level of serum Hcy was connected with the phenomenon of slow flow in coronary arteries. Oxidative stress and endothelial destruction related to Hcy were suggested to be the leading cause of slow coronary flow [33, 34]. Likewise, Hcy was shown to be associated with augmented arterial resistance in patients with internal carotid stenosis [36]. The altered hemodynamics may attribute to the remodeling of arterials with the effect of HHcy. Sun et al. reported that the flow velocity of internal carotid arteries and vertebral arteries was inversely correlated with Hcy quartiles in healthy participants, despite the parameters were not significantly different after the adjustments [37]. The difference of conclusions may be related to the inaccuracy of the data collected through carotid Doppler ultrasound, instead of transcranial Doppler sonography (TCD). As described, high-flow and low-resistance arterial blood flows directly into the venous system under the conditions of insufficient intervening capillaries in bAVMs, which carries a high risk of hemorrhage [1]. In this study, we revealed that the probability of hemorrhage was decreased in bAVM patients with higher Hcy. Therefore, we supposed that hemodynamic abnormalities induced by Hcy play an important role in the pathogenesis of bAVMs' rupture. The present results highlighted the correlation between Hcy and hemorrhage in bAVMs.

Specific morphological and angioarchitectural characteristics were proved to be related to hemorrhage of brain AVMs. The SM grading scale is used as the most common classification system in bAVMs, consisting of three anatomical factors (size of nidus, relative position of eloquent location, and patterns of draining veins) [38]. The five-classified grading system is a powerful tool for predicting operative risks based on the radiological data. In the current study, the maximum diameter of bAVMs was the independent predictor for rupture. In accordance with the present results, previous studies have illustrated that the incidence of subsequent hemorrhage increased with decreasing the size of lesions in untreated bAVMs [39, 40]. A small size of bAVMs (<3 cm) were more likely to present with bleeding compared to the larger lesions. In terms of other characteristics of bAVMs, no significant relationship between deep venous drainage or eloquent location and hemorrhage was evident in the present analyses. However, these variables were considered to be the key predictors for outcomes in previous studies [38]. The negative finding may be limited by the small sample size of enrolled participants.

There are several limitations to our study. First, this was a retrospective study with relatively small sample size, and it was difficult to avoid selection bias. Second, the diet patterns and supplements of folic acid or vitamins were not taken into consideration, and the variables may influence the metabolism of Hcy and affect the results. Third, there was a lack of data regarding levels of MMP-9 and hemodynamics parameters owing to the retrospective design; the direct relation between hemodynamic status and Hcy would be concluded with the support of evidence generated from TCD or DSA hemodynamic evaluation. Fourth, the clinical data derived from a high-volume single institution may present tentative associations between risk factors and bAVMs. Further prospective studies comparing Hcy before and after hemorrhage are required to investigate the predictive value for bAVM ruptures.

## 5. Conclusions

In conclusion, our results indicated that the Hcy levels were significantly associated with the risk of rupture in bAVM patients. A higher concentration of Hcy carried a lower prevalence of hemorrhage. It is recommended to monitor the factor in patients with bAVMs, which may facilitate the guidance of subsequent nutritional management strategies.

## Figures and Tables

**Table 1:** Baseline characteristics of bAVM patients.

Variables	Total (*n* = 143)	Unruptured (*n* = 69)	Ruptured (*n* = 74)	*P* value
Age, y, mean (SD)	29.6 (14.0)	31.0 (13.1)	28.2 (14.6)	0.221
Sex, male (%)	79 (55.2)	37 (53.6)	42 (56.8)	0.706
Medical history (%)				
Hypertension	9 (6.3)	2 (2.9)	7 (9.5)	0.204
Diabetes mellitus	3 (2.1)	1 (1.4)	2 (2.7)	1.000
Hyperlipidemia	1 (0.7)	0 (0)	1 (1.4)	1.000
Cigarette smoking	26 (18.2)	12 (17.4)	14 (18.9)	0.813
Alcohol drinking	16 (11.2)	7 (10.1)	9 (12.2)	0.702
Prior treatments (%)				
Embolization	21 (14.7)	7 (10.1)	14 (18.9)	0.139
Radiosurgery	6 (4.2)	1 (1.4)	5 (6.8)	0.244
Microsurgery	5 (3.5)	2 (2.9)	3 (4.1)	1.000
Primary symptom (%)				
Hemorrhage	74 (51.7)	0 (0)	74 (100.0)	<0.001^∗^
Seizure	30 (21.0)	25 (36.2)	5 (6.8)	<0.001^∗^
Neurological dysfunction	29 (20.3)	15 (21.7)	14 (18.9)	0.675
Admission mRS > 2 (%)	15 (10.5)	1 (1.4)	14 (18.9)	<0.001^∗^
AVM characteristics				
Spetzler-Martin grade (%)				0.149
I-II	81 (56.6)	34 (49.3)	47 (63.5)	
III	42 (29.4)	22 (31.9)	20 (27.0)	
IV-V	20 (14.0)	13 (18.8)	7 (9.5)	
Maximum diameter, cm, median (IQR)	3.4 (2.5-4.6)	3.9 (3.0-5.1)	2.9 (2.3-4.1)	<0.001^∗^
Volume, cm^3^, median (IQR)	8.6 (4.4-25.0)	15.8 (6.2-33.7)	6.0 (3.5-15.3)	<0.001^∗^
Deep location (%)	33 (23.1)	16 (23.2)	17 (23.0)	0.976
Eloquent location (%)	72 (50.3)	34 (49.3)	38 (51.4)	0.804
Deep venous drainage (%)	43 (30.1)	21 (30.4)	22 (29.7)	0.927
Associated aneurysms (%)	21 (14.7)	10 (14.5)	11 (14.9)	0.950
Clinical features, mean (SD)				
Heart rate, bpm	80 (10)	80 (9)	81 (11)	0.405
SBP, mm Hg	120 (15)	120 (13)	120 (17)	1.000
DBP, mm Hg	77 (10)	76 (10)	78 (11)	0.434
BMI, kg/m^2^	22.6 (4.4)	23.2 (4.4)	22.0 (4.4)	0.099
Laboratory results, median (IQR)				
Glucose, mmol/L	4.6 (4.3-5.0)	4.6 (4.3-4.8)	4.6 (4.4-5.1)	0.141
Albumin, g/L	43.9 (41.3-46.0)	44.4 (41.6-46.1)	43.5 (41.0-45.9)	0.511
Creatinine, *μ*mol/L	58.6 (46.6-68.5)	59.5 (49.7-70.9)	56.2 (45.2-67.8)	0.109
UA, *μ*mol/L	314.0 (257.7-366.5)	324.5 (280.8-374.8)	294.0 (241.8-357.1)	0.032^∗^
TC, mmol/L	4.3 (3.6-4.8)	4.3 (3.8-4.9)	4.2 (3.5-4.8)	0.468
TG, mmol/L	1.0 (0.7-1.5)	1.1 (0.8-1.6)	0.9 (0.7-1.5)	0.130
HDL-C, mmol/L	1.2 (1.0-1.5)	1.2 (1.0-1.4)	1.2 (1.1-1.5)	0.701
LDL-C, mmol/L	2.6 (2.0-3.2)	2.8 (2.1-3.5)	2.5 (1.9-3.1)	0.080
ApoA, g/L	1.3 (1.1-1.4)	1.3 (1.1-1.4)	1.3 (1.1-1.4)	0.588
ApoB, g/L	0.8 (0.7-1.0)	0.9 (0.7-1.1)	0.8 (0.7-0.9)	0.211
Hcy, *μ*mol/L	12.4 (9.9-16.2)	13.3 (10.4-18.9)	11.7 (9.7-14.6)	0.057
HHcy (%)	45 (31.5)	28 (40.6)	17 (23.0)	0.023^∗^

bAVM: brain arteriovenous malformation; SD: standard deviation; mRS: modified Rankin Scale; IQR: interquartile range; SBP: systolic blood pressure; DBP: diastolic blood pressure; BMI: body mass index; UA: uric acid; TC: total cholesterol; TG: triglyceride; HDL-C: high-density lipoprotein cholesterol; LDL-C: low-density lipoprotein cholesterol; ApoA: apolipoprotein A; ApoB: apolipoprotein B; Hcy: homocysteine; HHcy: hyperhomocysteinemia. ^∗^*P* < 0.05, significant difference.

**Table 2:** Characteristics of bAVM patients according to Hcy quartiles.

Variables	Hcy quartiles^a^, *μ*mol/L					*P* trend
	All (*n* = 143)	Q1 (*n* = 36)	Q2 (*n* = 35)	Q3 (*n* = 36)	Q4 (*n* = 36)	
Age, y, mean (SD)	29.6 (14.0)	23.8 (16.0)	30.2 (13.7)	35.4 (12.8)	28.9 (10.9)	0.005^∗^
Sex, male (%)	79 (55.2)	11 (30.6)	17 (48.6)	22 (61.1)	29 (80.6)	<0.001^∗^
Medical history (%)						
Hypertension	9 (6.3)	1 (2.8)	2 (5.7)	4 (11.1)	2 (5.6)	0.448
Diabetes mellitus	3 (2.1)	0 (0)	1 (2.9)	1 (2.8)	1 (2.8)	0.438
Hyperlipidemia	1 (0.7)	0 (0)	0 (0)	1 (2.8)	0 (0)	0.657
Cigarette smoking	26 (18.2)	1 (2.8)	6 (17.1)	10 (27.8)	9 (25.0)	0.007^∗^
Alcohol drinking	16 (11.2)	0 (0)	4 (11.4)	8 (22.2)	4 (11.1)	0.060
Primary symptom (%)						
Hemorrhage	74 (51.7)	21 (58.3)	20 (57.1)	19 (52.8)	14 (38.9)	0.093
Seizure	30 (21.0)	4 (11.1)	6 (17.1)	9 (25.0)	11 (30.6)	0.029^∗^
Neurological dysfunction	29 (20.3)	8 (22.2)	7 (20.0)	8 (22.2)	6 (16.7)	0.629
Admission mRS > 2 (%)	15 (10.5)	9 (25.0)	1 (2.9)	2 (5.6)	3 (8.3)	0.037^∗^
AVM characteristics						
Spetzler-Martin grade (%)						0.660
I-II	81 (56.6)	17 (47.2)	23 (65.7)	20 (55.6)	21 (58.3)	
III	42 (29.4)	13 (36.1)	8 (22.9)	12 (33.3)	9 (25.0)	
IV-V	20 (14.0)	6 (16.7)	4 (11.4)	4 (11.1)	6 (16.7)	
Maximum diameter, cm, median (IQR)	3.4 (2.5-4.6)	4.0 (2.6-4.7)	3.0 (2.5-4.4)	3.0 (2.3-3.9)	3.7 (2.6-5.1)	0.183
Volume, cm^3^, median (IQR)	8.6 (4.4-25.0)	10.9 (5.2-29.7)	7.2 (5.0-19.3)	8.2 (3.2-17.7)	13.9 (4.3-31.8)	0.239
Deep location (%)	33 (23.1)	8 (22.2)	7 (20.0)	8 (22.2)	10 (27.8)	0.549
Eloquent location (%)	72 (50.3)	19 (52.8)	18 (51.4)	19 (52.8)	16 (44.4)	0.526
Diffusive nidus (%)	68 (47.6)	21 (58.3)	17 (48.6)	10 (27.8)	20 (55.6)	0.434
Deep venous drainage (%)	43 (30.1)	12 (33.3)	8 (22.9)	13 (36.1)	10 (27.8)	0.916
Associated aneurysms (%)	21 (14.7)	6 (16.7)	5 (14.3)	6 (16.7)	4 (11.1)	0.588

bAVM: brain arteriovenous malformation; Hcy: homocysteine; SD: standard deviation; mRS: modified Rankin Scale; IQR: interquartile range. ^∗^*P* < 0.05, significant difference. ^a^Serum levels of Hcy in quartiles: Q1, <10.0 *μ*mol/L; Q2, 10.0-12.3 *μ*mol/L; Q3, 12.4-16.1 *μ*mol/L; and Q4, >16.1 *μ*mol/L.

**Table 3:** Logistic regression analysis on the risk of hemorrhage.

Variables	Univariate analysis			Multivariate analysis		
	OR	95% CI	*P* value	OR	95% CI	*P* value
Age	0.985	0.962-1.009	0.220	0.980	0.954-1.007	0.154
Male sex	1.135	0.587-2.196	0.707	1.565	0.686-3.568	0.287
Medical history						
Hypertension	3.500	0.701-17.467	0.127			
Diabetes mellitus	1.889	0.167-21.311	0.607			
Cigarette smoking	1.108	0.473-2.598	0.813	0.974	0.325-2.914	0.962
Alcohol drinking	1.226	0.430-3.495	0.703			
Prior treatments						
Embolization	2.067	0.780-5.475	0.144	2.848	0.868-9.347	0.084
Radiosurgery	4.928	0.561-43.286	0.150			
Microsurgery	1.415	0.229-8.737	0.708			
AVM characteristics						
Maximum diameter	0.668	0.519-0.859	0.002	0.634	0.479-0.839	0.001^∗^
Deep location	1.012	0.465-2.205	0.976	1.271	0.507-3.187	0.609
Eloquent location	1.087	0.564-2.094	0.804	1.230	0.561-2.698	0.605
Deep venous drainage	0.967	0.473-1.977	0.927	0.914	0.391-2.135	0.836
Associated aneurysms	1.030	0.408-2.603	0.950	1.184	0.415-3.380	0.753
Clinical features						
Heart rate	1.014	0.981-1.049	0.402			
SBP	1.000	0.978-1.022	>0.999			
DBP	1.013	0.981-1.046	0.432			
BMI	0.937	0.867-1.013	0.102			
Laboratory results						
Glucose	1.510	0.965-2.362	0.071			
Albumin	0.976	0.886-1.074	0.614			
Creatinine	0.978	0.957-1.000	0.051			
UA	1.000	0.999-1.001	0.964			
TC	0.912	0.638-1.305	0.616			
TG	1.052	0.764-1.449	0.756			
HDL-C	1.819	0.851-3.886	0.123			
LDL-C	0.724	0.493-1.064	0.100			
ApoA	0.916	0.272-3.089	0.888			
ApoB	0.404	0.081-2.012	0.269			
Hcy	0.965	0.934-0.998	0.038	0.956	0.920-0.993	0.021^∗^

OR: odds ratio; CI: confidence intervals; AVM: arteriovenous malformation; SBP: systolic blood pressure; DBP: diastolic blood pressure; BMI: body mass index; UA: uric acid; TC: total cholesterol; TG: triglyceride; HDL-C: high-density lipoprotein cholesterol; LDL-C: low-density lipoprotein cholesterol; ApoA: apolipoprotein A; ApoB: apolipoprotein B; Hcy: homocysteine. ^∗^*P* < 0.05, significant difference.

## Data Availability

The data used to support the findings of this study are available from the corresponding author upon request.
